# Canine laryngotracheal plasma cell tumors: Ten cases and literature review

**DOI:** 10.1177/03009858251331115

**Published:** 2025-04-11

**Authors:** Kathleen R. Mulka, Deborah Gillette, Amy C. Durham, Elizabeth A. Mauldin

**Affiliations:** 1 University of Pennsylvania, Philadelphia, PA

**Keywords:** canine, dog, extramedullary plasma cell tumor, laryngotracheal, plasmacytoma

## Abstract

Canine extramedullary plasma cell tumors (EMPs) most commonly arise in the skin, oral cavity, rectum, and colon. This retrospective study describes the histopathological and immunohistochemical characteristics and associated clinical signs and outcomes of laryngeal and tracheal EMPs in dogs. Five tracheal and 5 laryngeal EMPs were diagnosed at the Penn Vet Diagnostic Laboratory. Clinical information was obtained via submission forms and follow-up questionnaires. All dogs were male (9 castrated), 7 to 15 years old, and of different breeds. Neoplasms were composed of well-differentiated (n = 6) or moderately differentiated (n = 4) neoplastic plasma cells arranged in sheets, cords, and packets. All neoplasms labeled positively for MUM-1, negatively for PAX5, and were variably CD20- and CD79b-positive. There was no recurrence or disease progression 2 months to 7 years post biopsy in 6/9 cases. Results suggest that surgical resection can result in positive outcomes. Further studies are needed to identify factors leading to progression of this disease.

Canine extramedullary plasma cell tumors (EMP; also referred to as plasmacytoma) are derived from the expansion of a clonal population of neoplastic plasma cells outside of the bone marrow. Canine EMPs are most frequently located in skin, oral cavity, rectum, and colon. Sites less commonly reported include the stomach, spleen, genitalia, eye, uterus, liver, third eyelid, intracranial sites, larynx, and trachea.^5,20^ Most cutaneous and oral cavity EMPs are benign, and complete surgical excision is typically curative.^5^ A portion of oral EMP may be locally invasive and develop progressive disease or local recurrence.^5^ Alimentary EMPs are more commonly associated with metastasis to regional lymph nodes, and solitary osseous plasmacytomas are likely to progress to systemic multiple myeloma.^20^ Few single case reports describe tracheal and laryngeal plasma cell tumors in dogs.^1,2,4,6,15,22^ This is an uncommon location for this neoplasm, and the clinical behavior of this condition is not known. The objectives of this study were to describe the histopathological characteristics and immunophenotype of plasma cell tumors of the larynx and trachea, report clinical outcomes, and review the current literature on canine laryngotracheal EMPs.

The anatomic pathology database of the Penn Vet Diagnostic Laboratory was searched from January 2005 to April 2024 for submissions from the trachea or larynx. Cases containing “plasma cell tumor” or “plasmacytoma” in the diagnosis were included. Hematoxylin and eosin-stained sections from identified cases were reviewed by a board-certified anatomic pathologist (KRM). Neoplastic cells were evaluated for differentiation, anisocytosis/anisokaryosis, and nuclear atypia as outlined in Evenhuis et al.^5^ Immunohistochemistry for CD3 (Bio-Rad, MCA1477T), MUM-1 (Agilent (DAKO), M7259), CD79b (Cell Signaling Technologies #96024), CD20 (Thermo Scientific, RB9013P), and PAX5 (CST #12709) was performed. Formalin-fixed paraffin-embedded tissues were cut into 5-µm-thick sections and mounted on ProbeOn slides (ThermoFisher Scientific). Immunohistochemistry (IHC) was performed on a Leica BOND RXm automated platform using a Bond Polymer Refine Detection kit (Leica, DS9800 Allendale, NJ, USA) (Supplemental Table S1). Positive controls were canine lymph node for all antibodies (Supplemental Fig. S1). Negative controls were an irrelevant isotype-matched rat (CD3), mouse (MUM-1), or rabbit (CD79b, CD20, and PAX5) monoclonal antibody. Clinical information was obtained through pathology submission forms, medical records, questionnaires from referring veterinarians, and owner interviews.

From January 2005 to April 2024, 10 cases matched the inclusion criteria. All cases were male (1 intact, 9 castrated). The age at initial onset ranged from 7 to 15 years. There was one each of the following breeds/crosses: Maltese, Labrador retriever, beagle, golden retriever, goldendoodle, English springer spaniel, Jack Russell terrier, Yorkshire terrier, and 2 mixed breed dogs.

Eight dogs exhibited clinical signs associated with the mass, including cough, wheezing, inspiratory stridor, respiratory distress, hypersalivation, halitosis, and hemoptysis. In 2 cases, the mass was an incidental finding. Masses were identified via sedated oral exam or on intubation for a surgical procedure (3/10), fluoroscopy (1/10), radiographs (5/10), and/or computed tomography imaging (4/10). Grossly, masses were pink with smooth margins occluding up to 75% of the tracheal lumen. Four of the tracheal masses were associated with the dorsal tracheal membrane. Masses were surgically resected (3/10), submitted as an incisional biopsy (3/10), marginally excised using biopsy graspers (1/10), or debulked using a snare and electrocautery via endoscopy (3/10) (Supplemental Table S2).

The neoplasms were composed of densely cellular populations of round cells arranged in sheets and cords (Fig. 1a–d). In 6/10 cases, the neoplastic cells formed packets with a moderate fibrovascular stroma (Fig. 1d). Neoplastic cells had small to moderate amounts of variably vacuolated eosinophilic cytoplasm. Nuclei were round to oval with coarsely clumped chromatin and occasional peripheralized nuclei with perinuclear clear zones (Golgi zones) (Fig. 1c, d). Four of 10 neoplasms were considered moderately differentiated, and 6/10 were considered well-differentiated.^5^ Karyomegalic, bi-nucleate, and tri-nucleate cells were observed in all cases to varying degrees. The mitotic count ranged from 1 to 67 in 10 high-power fields (2.37mm^2^) (Supplemental Table S3). Amyloid was present in 3 cases (cases 4, 9, and 10), which had lower cellularity compared with cases without amyloid (Fig. 1e). Neoplastic cells extended to the surgical margins in all cases.

**Figure 1.**
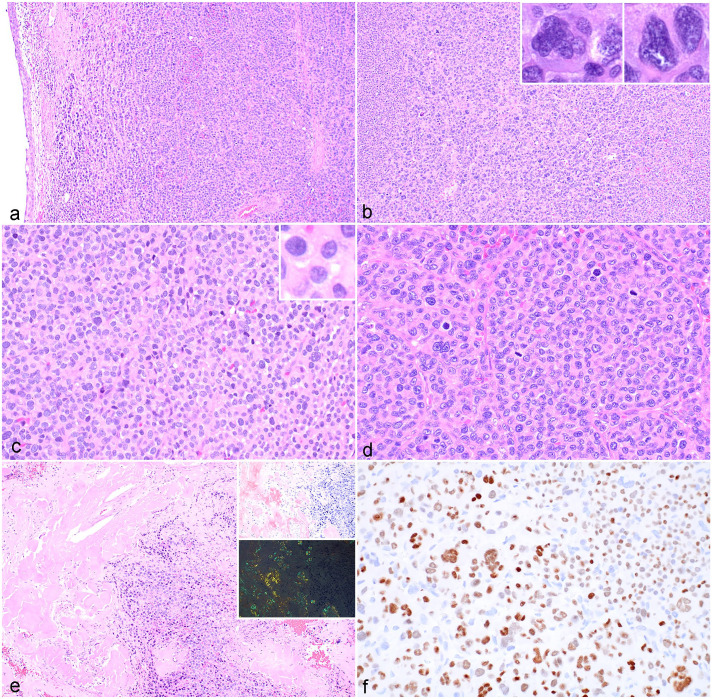
Plasma cell tumor, trachea, dog. (a) Neoplastic plasma cells arranged in sheets and cords expand the tracheal submucosa. Case 6. Hematoxylin and eosin (HE). (b) Numerous karyomegalic and multinucleated neoplastic cells are noted. Insets: higher magnification of karyomegalic and multinucleated cells. Case 6. HE. (c) Well-differentiated neoplastic plasma cells have moderate amounts of eosinophilic cytoplasm, round nuclei with coarsely clumped chromatin, and a variably prominent perinuclear clear zone, with scattered karyomegaly and multinucleation. Inset: higher magnification of cells with a prominent perinuclear clear zone. Case 7. HE. (d) Neoplastic plasma cells are arranged in packets separated by a moderate fibrous stroma. Case 6. HE. (e) Amyloid is present in a subset of cases. Case 4. HE. Insets: amyloid stains red-orange with Congo red (top inset) and demonstrates apple-green birefringence when viewed with polarized light (bottom inset). Case 6. Congo red. (f) Neoplastic cells have strong positive immunoreactivity to MUM-1. Case 6. MUM-1 immunohistochemistry.

Ten of 10 neoplasms demonstrated positive nuclear immunoreactivity to MUM-1 in greater than 50% of neoplastic cells, which varied in intensity (Fig. 1f). Four of 10 EMPs had positive cytoplasmic immunoreactivity to CD79b. Two of 10 EMPs had positive cytoplasmic immunoreactivity to CD20. All were negative for PAX5. One of 10 EMPs contained a population of cells that had strong cytoplasmic immunoreactivity for CD3 (Supplemental Table S3). Polymerase chain reaction for antigen receptor rearrangements (PARR) testing was performed on this case (Veterinary Diagnostics, Davis, CA) and revealed clonal results for IgH2 and IgH3 and pseudoclonal and polyclonal results for T-cell receptor gamma (TRG) (A), TRG (B), and TRG(C).

Follow-up information was available for 9/10 cases and summarized in Supplemental Table S2. Case 1 was lost to follow-up. Three animals (cases 2, 7, and 8) were deceased. In case 2, the patient was euthanized 2 weeks after biopsy due to worsening clinical signs associated with the tracheal EMP. Autopsy revealed a 2.1 cm × 0.9 cm × 0.6 cm, firm, tan, poorly demarcated, multinodular mass arising from the dorsolateral aspect of the trachea 7.5 cm distal to the larynx, occluding approximately 30% of the trachea. There was no evidence of metastasis. Case 7 had radiosurgery with no recurrence in 7 years and was euthanized due to unrelated causes. Case 8 was reported to have clinical disease progression and was euthanized 3 months post-biopsy. Six animals were alive at last follow-up. Case 3 had an occasional non-productive cough and no other respiratory signs 6 months post-biopsy. This patient had several other ailments and was undergoing palliative care. The patient is presumed to have died shortly after this visit and the association between the clinical decline with the EMP is unclear. The sample from case 4 was obtained via alligator punch forceps and a palliative permanent tracheostomy was performed. Follow-up was obtained 2 months post-biopsy with no progression of disease, and the patient was subsequently lost to follow-up. Cases 5, 6, 9, and 10 had no reported recurrence or clinical progression at follow-up times varying from 3 to 14 months post-biopsy.

A literature search identified 8 individual case reports of dogs with EMPs of the larynx (2) or trachea (6).^1,2,4,6,9,15,21,22^ There were 5 males (1 intact, 4 castrated) and 3 spayed females, representing a variety of breeds. The age of onset ranged from 3 to 11 years. Clinical signs included dyspnea, stridor, cough, collapse/syncope, labored breathing, dysphonia, choking, and hemoptysis. The masses were pedunculated, pink, and had a smooth surface. Four of 6 tracheal masses originated from the dorsal tracheal membrane, 1/6 was attached to the ventral aspect of the trachea, and 1/6 originated from the left side of the trachea. The masses obstructed 70%–90% of the tracheal lumen. In the 2 laryngeal masses, 1 originated in the cranial dorsal surface of the epiglottis and the other from the left ventrolateral vocal fold.

The case reports describe similar histologic findings consisting of neoplastic populations of round cells tightly packed in a thin fibrovascular stroma. Neoplastic cells had eosinophilic and granular cytoplasm with a pale paranuclear clear zone and round, eccentrically located nuclei with stippled chromatin. Bi- or multinucleated cells were observed. Mitotic counts, when available, were 1 to 3 mitotic figures in 10 high-power fields (area not defined in millimeters). Immunohistochemistry was performed in some reports. In one study, neoplastic cells were positive for MUM-1 and negative for chromogranin A.^9^ In another study, neoplastic cells were MUM-1-positive and no other markers were run.^4^ In 2 studies, neoplastic cells were negative for CD3 and positive for CD79a.^6,22^

Within the previously published case reports, all 6 tracheal EMPs were surgically excised. One case described mass removal without resection of additional tracheal tissue. This animal was symptom-free 18 months post-surgery.^21^ Four of 6 dogs underwent tracheal ring resection and anastomosis. Of these cases, 3 reported no evidence of recurrence at 3 months^2,15^ and 9 months^9^ post-surgery. The fourth case was initially diagnosed as nodular immunocyte-derived (AL) amyloidosis; 17 months post-surgery, a tracheal plasmacytoma with hepatic metastasis was diagnosed at autopsy. This was reported as a case of progression from nodular amyloidosis to plasmacytoma with metastasis.^1^ One EMP was excised endoscopically by laser ablation; however, the neoplasm recurred and partial tracheal ring resection was performed 7 weeks after the initial surgery. One year after surgery the patient was free of clinical signs relating to the tracheal mass.^4^ Following biopsy of the 2 laryngeal masses, 1 case underwent radiation therapy followed by systemic chemotherapy and was alive without recurrence 30 months after the initial presentation and 12 months after stopping chemotherapy.^6^ The other laryngeal EMP received chemotherapy only and had recurrence after 6 months of chemotherapy. An autopsy revealed EMP at the initial site with no evidence of metastasis.^22^

In cats, 2 reports described EMPs involving components of the respiratory tract.^13,18^ One case report documented an invasive EMP with involvement of the frontal sinus, nasal cavity, soft palate, larynx, trachea, lungs, and multiple lymph nodes. In the other case, an EMP was present within the right nasal cavity and frontal sinus without involvement of other tissues.^13^ In both instances, the EMP caused severe respiratory signs and resulted in the euthanasia of the animal.^13,18^

In humans, EMP most commonly arise in the upper respiratory tract (nasal cavity, paranasal sinuses, nasopharynx, and larynx), while EMP of the trachea is rare.^3^ Recurrence and progression to multiple myeloma are reported in a minority of cases in humans with EMP of the upper aerodigestive tract, and therefore, long-term follow-up is recommended.^17^

The current study is the first immunohistochemical characterization of canine laryngotracheal EMP. B-cell markers including CD79b, CD20, and PAX5 were assessed in addition to CD3 (T-cell marker) and MUM-1 (plasma cell marker). CD79b is associated with the B-cell receptor, is involved in initiation of B-cell receptor signaling and B-cell activation, and is expressed on immunoglobulin-positive B cells.^8^ CD20 is a transmembrane calcium channel that is expressed in mature B-cells.^8^ Plasma cell differentiation is promoted by downregulation of PAX5, a transcription factor that is necessary for B-cell development.^7,14^ While a portion of EMP may be immunopositive for CD79b and CD20, they are expected to be negative for PAX5 and CD3.^10^

Consistent with EMP in other locations,^7,10,14^ all neoplasms in our cases were MUM-1-positive, variably positive for CD20 and CD79, and negative for PAX5 and CD3. MUM-1 is commonly used in the diagnosis of plasma cell neoplasms in dogs. However, positive immunoreactivity to MUM-1 has also been reported in cutaneous histiocytomas and in subsets of canine lymphomas and lymphoproliferative disorders.^7,10,11,16^ MUM-1 should be run in conjunction with other round cell markers such as IBA-1, CD3, CD79, and CD20. In cases of overlapping immunopositivity, PARR is useful to determine clonality of the neoplastic population.^7,19^

In cases with amyloid deposition, the neoplastic cellularity can be low. Two case reports of nodular respiratory tract amyloidosis have been documented in dogs.^1,12^ Both cases describe infiltration of low numbers of lymphocytes and plasma cells amid the extracellular depositions of amyloid.^1,12^ In one case, clonality of plasma cells was assessed on an initial biopsy and determined to be polyclonal. In subsequent autopsy, the lesion had progressed, and a clonal population of plasma cells was isolated.^1^ In biopsies of nodular lesions of the trachea or larynx composed predominantly of amyloid, EMP should be considered regardless of the cellularity of the sample.

In summary of the findings from the 10 cases described here, in conjunction with the 8 published case reports, the histopathologic findings of laryngotracheal EMPs are similar and may overlap with other round cell or neuroendocrine neoplasms. In these instances, immunohistochemistry and PARR testing are valuable diagnostic aids. A review of the available clinical outcomes results for this combination of cases suggest that surgical resection can result in positive outcomes or be curative. Recurrence or progression of disease was observed in a smaller subset of cases. When surgical removal was not attempted, clinical signs progressed necessitating euthanasia. Additional investigations into these neoplasms are warranted to further assess their clinical behavior and to identify factors leading to disease progression.

## Supplemental Material

sj-pdf-1-vet-10.1177_03009858251331115 – Supplemental material for Canine laryngotracheal plasma cell tumors: Ten cases and literature review Supplemental material, sj-pdf-1-vet-10.1177_03009858251331115 for Canine laryngotracheal plasma cell tumors: Ten cases and literature review by Kathleen R. Mulka, Deborah Gillette, Amy C. Durham and Elizabeth A. Mauldin in Veterinary Pathology
