# Supplementary material for: Canine laryngotracheal plasma cell tumors: Ten cases and literature review
Source: Vet Pathol. 2025 Apr 11;62(5):692–6. doi: 10.1177/03009858251331115 (PMC12314212; doi:10.1177/03009858251331115)
Supplement: sj-pdf-1-vet-10.1177_03009858251331115 – Supplemental material for Canine laryngotracheal plasma cell tumors: Ten cases and literature review [file sj-pdf-1-vet-10.1177_03009858251331115.pdf]

Supplemental Materials

Canine laryngotracheal plasma cell tumors: Ten cases and literature review

Kathleen R. Mulka, Deborah Gillette, Amy C. Durham, Elizabeth A. Mauldin

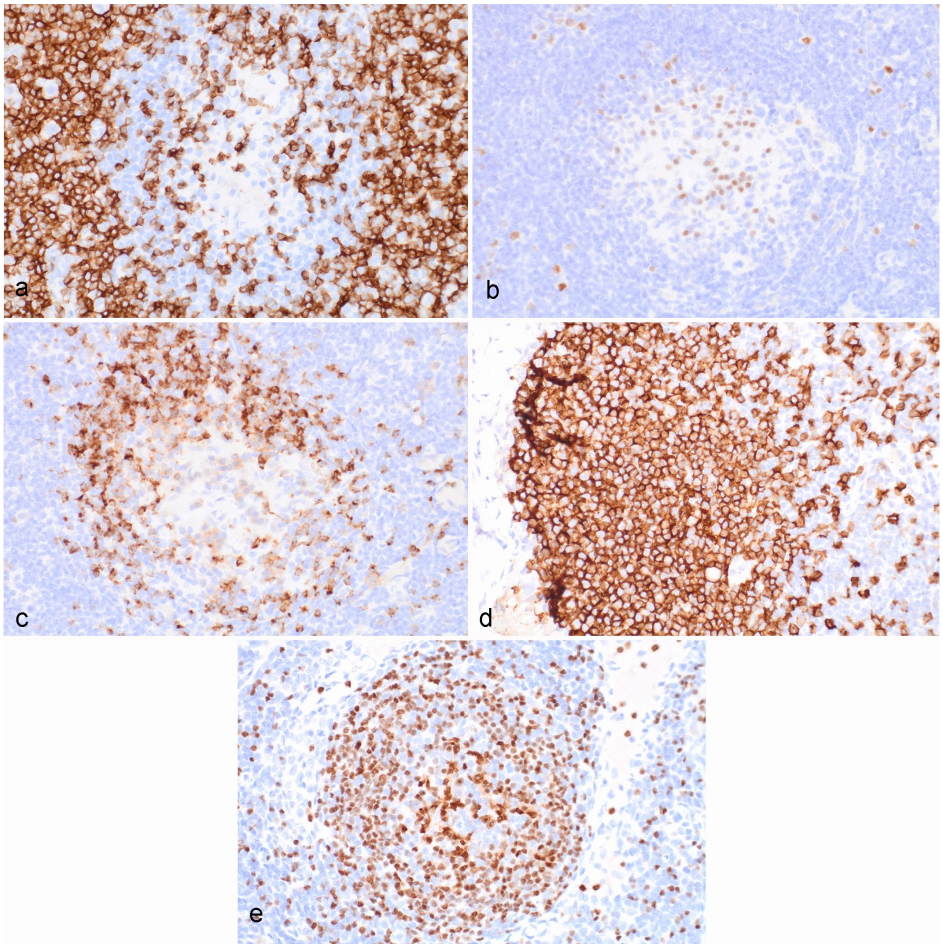

Supplemental Figure S1. Immunohistochemistry (IHC), lymph node, dog. Canine lymph node tissue was used as a positive control for IHC. Samples were labeled for **a)** CD3 **b)** MUM-1 **c)** CD79b **d)** CD20 and **e)** PAX 5.

Supplemental Table S1. Immunohistochemistry protocols.

	MUM-1	CD3	CD20	CD79b	PAX 5
Antibody	Agilent (DAKO), M7259	Bio-Rad, MCA1477T	Thermo Scientific, RB9013P	CST #96024	CST, #12709 (D7H5X)
Host species	Mouse	Rat	Rabbit	Rabbit	Rabbit
Antigen retrieval	ER2[a]	ER2	ER2	ER2	ER2
Dilution	1:50	1:600	1:500	1:300	1:100
Chromogen	DAB	DAB	DAB	DAB	DAB

[a] EDTA Based pH=9.0, Leica AR9640

Abbreviations: CST, Cell Signaling Technology; DAB, diaminobenzidine

Supplemental Table S2. Signalment and clinical information for 10 cases of canine laryngotreacheal plasma cell tumors.

Case	Breed	Sex	Age (Years)	Clinical Signs	Imaging Modality and Results	Location of the EMP	Biopsy Type	Treatment	Status at Last Follow-up [interval following biopsy diagnosis in months]
1	Maltese	MC	10	Elevated respiratory rate/effort; cough	Fluoroscopy: mass on the dorsal epiglottis	Right Arytenoid	Excisional; surgical resection	None	N/A [LTFU]
2	Beagle	MC	8	Cough/sneeze	Radiographs: mediastinal mass, collapse of cranial trachea; CT intraluminal tracheal mass	Trachea, arising from dorsal aspect	Incisional; endoscopy with gastroscope	None	Deceased due to tracheal neoplasm [0.5]
3	Labrador retriever	MC	10	Cough	CT: intraluminal tracheal mass	Trachea	Partial mass resection; endoscopy; 60-70% of mass debulked using snare	Tracheal stent placement at time of biopsy	Alive but undergoing palliative care for several other ailments; association to the tracheal EMP is unclear [6]
4	Mixed	M	11	Cough; respiratory distress	Radiographs: trachea mass	Trachea, arising from wall	Incisional; endoscopy with alligator punch forceps	Permanent tracheostomy at time of biopsy [palliative]	Alive with no progression of disease [2.25]
5	Mixed	MC	11	Cough; wheezing	Radiographs: mediastinal mass, cranial tracheal collapse; CT intraluminal tracheal mass	Trachea, dorsal aspect	Partial mass resection; endoscopy with snare and electrocautery	Tracheobronchial wall stent at time of biopsy	Alive with occasional cough [14]
6	Golden retriever	MC	7	Inspiratory stridor; trouble breathing	Radiographs: tracheal mass; CT: mass occluding 30% of tracheal lumen	Trachea, attached to dorsal membrane	Excisional; endoscopy with snare and electrocautery	None	Alive with no respiratory signs or progression of disease [10]
7	Goldendoodle	MC	8	Hypersalivation; halitosis	None	Epiglottis	Excisional; surgery	Base of excision and 0.75 margin tissue ablated with radiosurgery	Deceased due to other causes; no recurrence of disease [74]
8	English springer spaniel	MC	12	Hemoptysis	Radiographs: mass	Epiglottis	Wedge; surgery	None	Deceased due to progression of disease [3]
9	Jack Russel terrier	MC	15	None; incidental finding	None	Arytenoid Cartilage	Incisional; surgery	None	Alive with no respiratory signs or progression of disease [3]
10	Yorkshire terrier	MC	7	None; discovered on intubation for another procedure	None	Epiglottis	Marginally excised using biopsy graspers	None	Alive with no respiratory signs or progression of disease [3]

Abbreviations: EMP, extramedullary plasma cell tumor; MC, male castrated; N/A not applicable; LTFU, lost to follow-up; M, male; CT, computed tomagraphy

Supplemental Table S3. Histologic features and immunohistochemistry results of 10 canine laryngotracheal plasma cell tumors.

Case	Differentiation	Anisocytosis/ Anisokaryosis	Nuclear Atypia	Multinucleation (in 10 HPF, 2.37 mm^2)	Mitotic Count (in 10 HPF, 2.37 mm^2)	Amyloid	MUM-1	CD3	CD20	PAX5	CD79b
1	moderate	moderate	mild	12	23	no	+	-	+	-	-
2	moderate	moderate	mild	10	10	no	+	-	+	-	+
3	well	moderate	mild	12	3	no	+	-	-	-	-
4	moderate	moderate	mild	1	4	yes	+	-	-	-	-
5	well	moderate	mild	4	67	no	+	-	-	-	+
6	well	moderate	mild	7	65	no	+	-	-		+
7	well	moderate	mild	29	23	no	+	-	-	-	+
8	well	moderate	mild	3	30	no	+	-	-	-	-
9	well	mild	mild	1	0	yes	+	-	-	-	-
10	moderate	mild	mild	1	2	yes	+	-	-	-	+
